# Evaluation of non-thermal effect of microwave radiation and its mode of action in bacterial cell inactivation

**DOI:** 10.1038/s41598-021-93274-w

**Published:** 2021-07-07

**Authors:** Priyanka Shaw, Naresh Kumar, Sohail Mumtaz, Jun Sup Lim, Jung Hyun Jang, Doyoung Kim, Bidya Dhar Sahu, Annemie Bogaerts, Eun Ha Choi

**Affiliations:** 1 Plasma Bioscience Research Center, Department of Electrical and Biological Physics, Kwangwoon University, Seoul, 01897 Korea; 2 Research Group PLASMANT, Department of Chemistry, University of Antwerp, 2610 Wilrijk-Antwerp, Belgium; 3 Department of Pharmacology and Toxicology, National Institute of Pharmaceutical Education and Research, Guwahati, Guwahati, Assam 781101 India

**Keywords:** Biophysics, Microbiology, Energy science and technology

## Abstract

A growing body of literature has recognized the non-thermal effect of pulsed microwave radiation (PMR) on bacterial systems. However, its mode of action in deactivating bacteria has not yet been extensively investigated. Nevertheless, it is highly important to advance the applications of PMR from simple to complex biological systems. In this study, we first optimized the conditions of the PMR device and we assessed the results by simulations, using ANSYS HFSS (High Frequency Structure Simulator) and a 3D particle-in-cell code for the electron behavior, to provide a better overview of the bacterial cell exposure to microwave radiation. To determine the sensitivity of PMR, *Escherichia coli* and *Staphylococcus aureus* cultures were exposed to PMR (pulse duration: 60 ns, peak frequency: 3.5 GHz) with power density of 17 kW/cm^2^ at the free space of sample position, which would induce electric field of 8.0 kV/cm inside the PBS solution of falcon tube in this experiment at 25 °C. At various discharges (D) of microwaves, the colony forming unit curves were analyzed. The highest ratios of viable count reductions were observed when the doses were increased from 20D to 80D, which resulted in an approximate 6 log reduction in *E. coli* and 4 log reduction in *S. aureus.* Moreover, scanning electron microscopy also revealed surface damage in both bacterial strains after PMR exposure. The bacterial inactivation was attributed to the deactivation of oxidation-regulating genes and DNA damage.

## Introduction

Microwave-based disinfection technologies are becoming increasingly popular because of advancements in equipment consistency, reductions in unwanted disinfection byproducts, and household applications^1^. Microwaves are electromagnetic waves with frequency ranging from 0.3 to 300 GHz (i.e., wavelengths from 1 m to 1 mm)^1,2^. Microwaves are ionizing radiation that occur next to the infrared component of the electromagnetic spectrum^2^. However, the electric field is mainly responsible for heating^3^. Indeed, the physical laws of dielectric heating by microwaves and the effects of heat on biological systems are well known^4^. It is commonly thought that the inactivation of microorganisms is mainly caused by a rise in temperature following microwave exposure^5,6^, but the thermal effect of microwaves differs from that of conventional heating. More specifically, microwaves can also cause thermal effects, just like in conventional heating, but it occurs by another mechanism, i.e., by means of the dielectric property of molecules, more specifically, for polar substances, with shorter reaction times than for conventional heating. Conventional heating is slow and is introduced into the sample from the surface. It has been observed that microwaves can destroy microorganisms at temperatures lower than the thermal destruction point. In particular, cells of *S. aureus* irradiated by microwaves exhibited a greater metabolic imbalance than conventionally heated cells^7^. During microwave propagation, both thermal and non‐thermal effects can alter the intracellular components of the microorganisms. However, several researchers have attempted to find out if such radiation has a non-thermal effect on microorganisms^7,8^. Moreover, Rougier et al.^5^, showed that 2.45 GHz microwave exposure at 37 °C induced *Escherichia coli* membrane modification. The authors observed the release of intracellular proteins in bacterial suspensions and approximately 8% of permeabilized cells appeared after microwave exposure, while conventional heating at 37 °C did not induce any effect. Moreover, the results showed that conventional heating at 47 °C for 10 min or at 48 °C for 5 min was necessary to induce the same effects. Woo et al., reported that non-thermal microwave radiation in *Escherichia coli* and *Bacillus subtilis* cell suspensions resulted in a dramatic reduction of the viable counts, as well as an increase in the amounts of DNA and proteins released from the cells^9^.

There exist also other sterilisation methods based on electromagnetic fields, such as gamma irradiation^10^. However, gamma irradiation for sterilization is not always practical as it is very expensive, and actually it can damage the material^11^ because it contains very high energy. Recently, non-thermal cold atmospheric plasma (CAP) has shown good potential for sterilisation, e.g., in the context of food decontamination^12,13^, but CAP processing has been shown to affect the quality of the food products during treatment, as well as in storage^14,15^, so further studies are needed to improve the use of CAP in sterilization or food packaging. Moreover, the inactivation of bacterial spores by heavy ions resulted in inactivation (determined from loss of colony formers), mutagenesis (reversion to histidine prototrophy), and inhibition of DNA repair. It is still not clear however whether the repair systems are inactivated, or merely that heavy-ion lesions are less repairable^13,16^.

In addition to its use in domestic applications, microwaves have been applied in various other fields. Microwaves are applied primarily to achieve thermal effects for purposes such as food processing^17^, waste treatment^18^, moisture removal^19^, disinfection, sterilization^20^ and the inactivation of several microorganisms, including *Clostridium perfringens*, *Bacillus subtilis Salmonella*, *Listeria* spp., and mold spores. It has been reported that the bacteriophage PL-1, which is specific to *Lactobacillus casei*, is also sensitive to microwave radiation^21–24^.

Several studies have revealed that non-thermal pulsed microwave radiation (PMR) can influence the cellular metabolism without raising the temperature of the system. Nevertheless, the same absorption resonance and induced electromagnetic energy are used as when using microwave radiation for its thermal effects^9,25^. In recent years, non-thermal radiation has been also extensively used in our daily life and has become popular in the development of military equipment, imaging, and sensing for early stage tumor detection, blood clot/stroke detection^26–32^, and bacterial inactivation^9^.

Despite the wide use of this technology, the impact of PMR on microbial inactivation is not so clear, and a better understanding of the non-thermal effects (particularly biological effects) and the mode of action of PMR is urgently needed. Therefore, this study aims to examine the bacterial inactivation by microwave radiation at certain frequencies, at high energy and very short duration, and to elucidate the mechanism of microbial cell inactivation. We used an PMR device at a dominant frequency of 3.5 GHz with 60 ns pulse duration and varying numbers of electromagnetic discharges, to investigate their effects on two highly prevalent bacterial species, i.e., the gram-positive *Staphylococcus aureus* and gram-negative *Escherichia coli*, and we try to reveal the underlying mode of action. It should be kept in mind, however, that the frequency range of 1–4 GHz can significantly change the dielectric property of materials^33,34^. A significant number of publications reported an increased level of reactive oxygen species (ROS) after microwave exposure^35–38^. On the other hand, a few studies reported no effect of MW exposure on the ROS levels^39,40^. A few reviews revealed that 90% of all studies analyzing the ROS levels after MW exposure have reported ROS induction in different cell types^41,42^ and these increased levels of ROS are often associated with oxidative stress-induced cell death upon exposure to microwave radiation.

To understand the effect of PMR at various electromagnetic doses, we evaluated the output characteristics of PMR and, after optimizing the PMR device, we studied its effect on the morphological changes in bacterial cells, oxidative stress-mediated DNA damage, and antioxidant-related gene expression. Overall, this work is important for possible future applications of PMR technology at the industrial level.

## Experimental section

### Pulsed microwave radiation (PMR) generator

**Figure 1:**
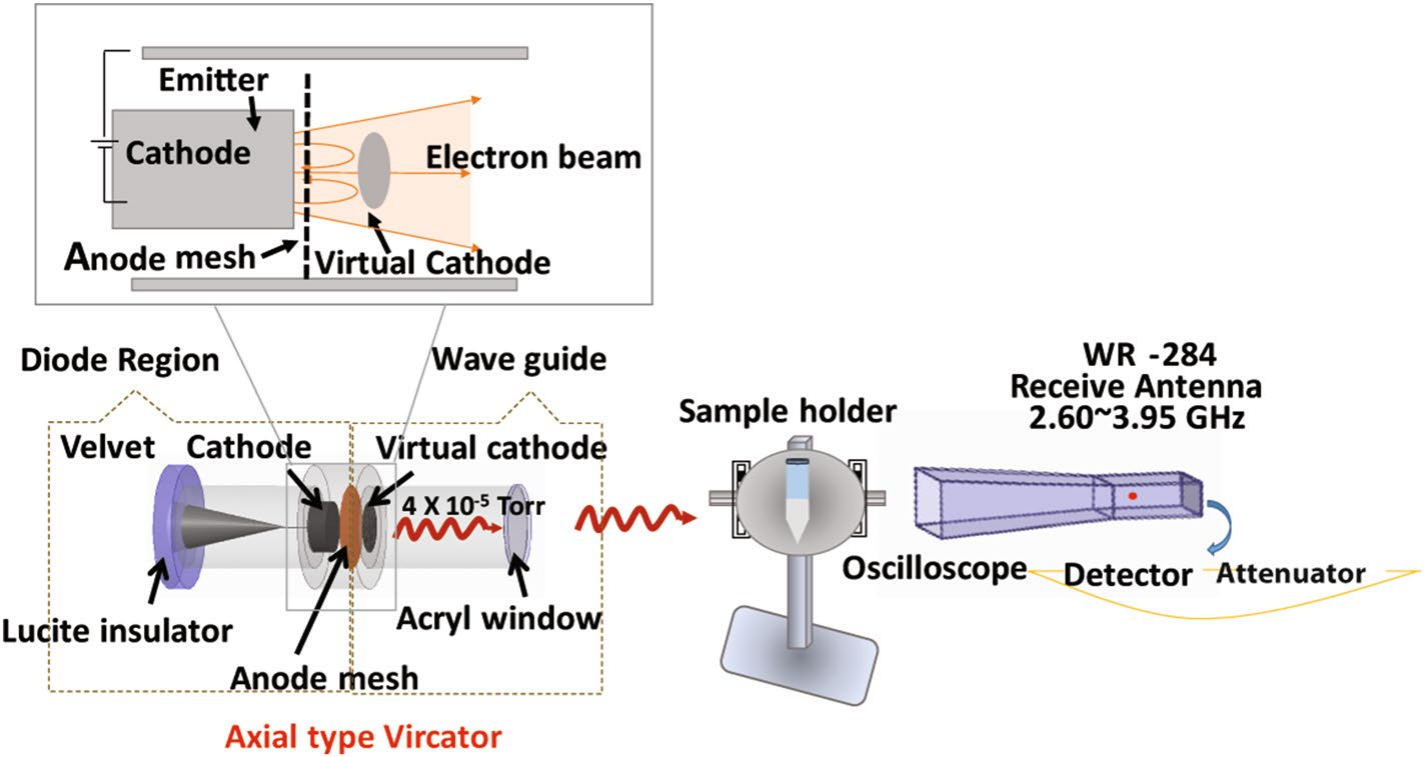
Schematic diagram of the non-thermal pulsed microwave radiation (PMR) device.

A PMR generator with an axial voltage of 600 kV, current of 88 kA, and 60 ns pulse duration was used. The vircator comprised three major components: a cathode, meshed metallic anode, and a waveguide with an inner diameter of 20 cm and a length of 25 cm. A schematic of the axial vircator is shown in Fig. 1. The vircator produces electrons from the cathode, composed of aluminum (10 cm in diameter), which are accelerated towards the meshed anode. The anode is transparent to allow most of the electron beam to pass through it. When the potential energy in the beam is higher than its kinetic energy, an electron cloud, known as a virtual cathode (VC), is formed behind the anode. After electrons are reflected between the cathode and the virtual cathode, an electromagnetic wave is generated^43,44^. The vacuum in the diode was maintained at 10^−5^ Torr and was generated 1 h prior to the experiment. To maintain a high vacuum inside the diode region, the end of the drift tube is bounded by a 1.5-cm-thick acryl window to allow the microwaves to easily propagate through the acryl material.

### Output characteristics of the PMR apparatus

**Figure 2:**
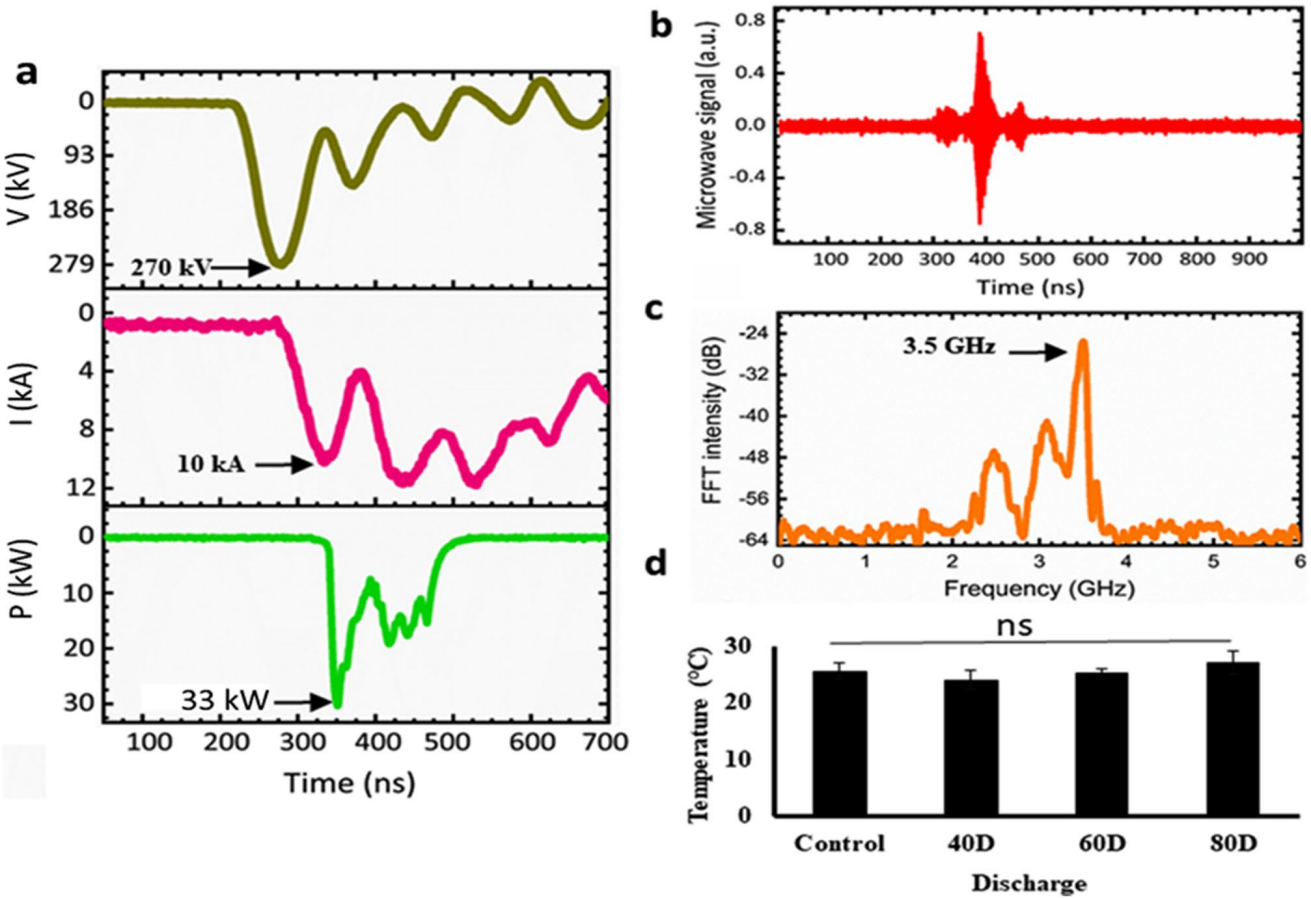
Physical characteristics of the PMR apparatus. (**a**) Typical waveform of the diode voltage, diode current, and microwave envelope signals, (**b**) microwave signal, (**c**) dominant frequency of the microwaves, and (**d**) temperatures of the saline after the different PMW exposure (the room temperature was 25 °C).

The microwave signal was detected using a horn antenna (WR-284) with a cutoff frequency of 2.14 GHz. All signals were recorded using a four-channel oscilloscope (Wave Master 8620A, LeCroy Corporation, Chestnut Ridge, NY, USA), as shown in Fig. 2, in an electromagnetically protected screen room. The microwave envelope signal was recorded by a crystal detector (Narda 4503A, Narda Miteq, Hauppauge, NY, USA). The magnitudes of the frequencies were calculated by fast Fourier transformation. Two home-made probes, not shown in Fig. 1, which we call C-dot and B-dot probes, were used to measure the diode voltage and the diode current, respectively^45^. The C-dot and B-dot probes are connected from the microwave generator to the four-channel oscilloscope.

After different microwave exposure, i.e., 40, 60, and 80 discharges, the temperature of the saline was measured using an Infrared (IR) camera (Fluke Ti100 Series Thermal Imaging Cameras, UK). The temperature was measured by placing the saline in the treatment room of 25 °C.

### In silico characterization of virtual cathode formation

**Figure 3:**
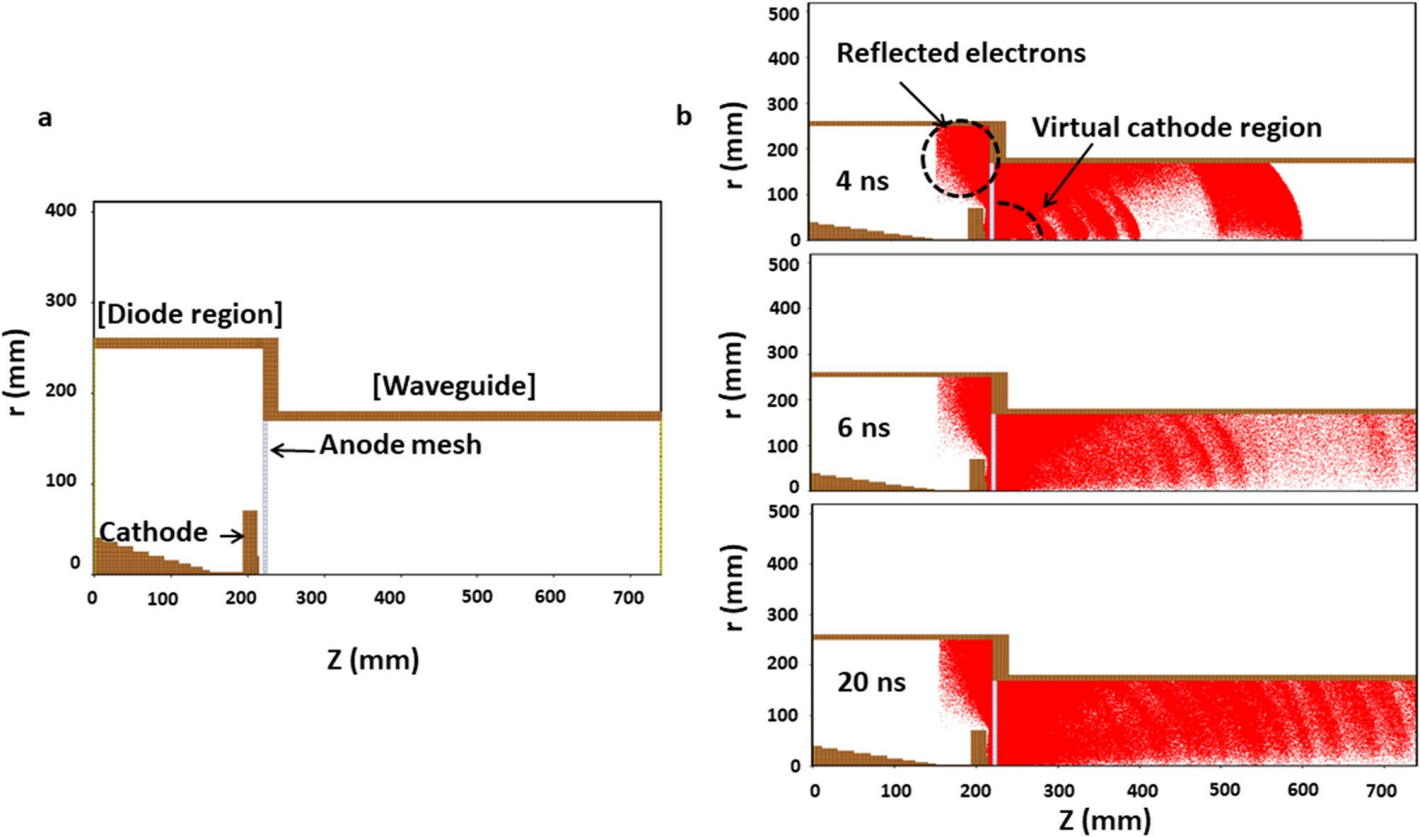
Results of the 3D-PIC simulations: Behavior of electrons inside the diode and formation of the virtual cathode during the simulation time. (**a**) Diode design of the axial-type vircator used in silico (Radius versus electron momentum). (**b**) Phase-space plot for the incremental behavior of electrons inside the diode region at 4, 6, and 20 ns.

The characteristics of virtual cathode formation and its dynamics were computationally investigated using the three-dimensional particle-in-cell code, called MAGIC^45,46^. We used 300 kV voltage and 10 kA current, based on the experiments. Furthermore, electrons were designed to be emitted from the cathode surface by using the MAGIC code command “EXPLOSIVE EMISSION” (as shown in Fig. 3). Previously, we reported that the PMR generated from virtual cathode in this experiment has coherent characteristics^46,47^, which is verified by MAGIC simulation code and experiments for the power intensity profile along the propagation direction. In addition, another study reported that this coherent characteristics is caused from the electrons that are dynamics at the virtual cathode^48^.

**Figure 4:**
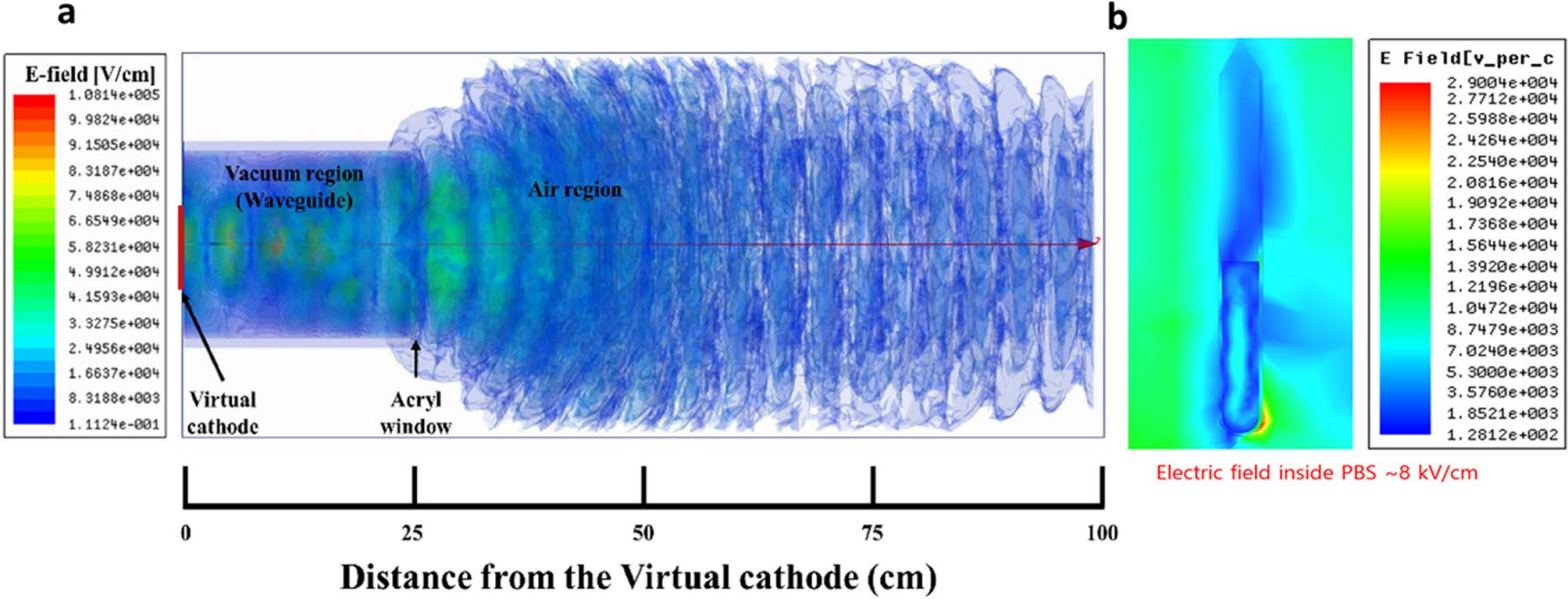
(**a**) Results of the HFSS simulations: electric field distributions in PMR from the virtual cathode to the outside environment (up to 100 cm); (**b**) electric field distribution with 8 kV/cm inside the PBS solution in falcon tube.

Furthermore, to optimize the position (i.e., where bacteria should be treated) to achieve the highest possible electric field on the axis of the waveguide for a given input power, we performed a full wave electromagnetic simulation outside the waveguide together with a vircator in the ANSYS HFSS (high frequency structure simulator) code (Fig. 4)^49^. In the HFSS electromagnetic simulation software, the parameters such as voltage, current and pressure used were all taken from experiments, and the setup was kept at the design value of the device.

### Chemicals

Luria Bertani agar, Luria Bertani broth, tryptic soy agar, and tryptic soy were purchased from MB cells (Seoul, Korea). Strains of wild–type *E. coli* (11775) and wild-type *S. aureus* 1621 were procured from the American Type Culture Collection (Manasses, VA, USA) and Korean Culture Center of Microorganisms (Seoul, South Korea). Genomic DNA was extracted using a DNA extraction kit (GeneAll, Exgene Cell SV MAXI, Seoul, Korea).

### Sample preparation

Wild-type *E. coli* and wild-type *S. aureus* were obtained from the stock culture in our laboratory^50,51^. *E. coli* was grown in Luria broth*,* and *S. aureus* was grown in nutrient medium. The bacteria were cultured in 500 mL liquid medium at 37 °C for 15 h on a rotary shaker (150 rpm) until they reached logarithmic growth. The cells were harvested by centrifugation and washed twice with a sterile 0.9% NaCl solution. The cell pellets were resuspended in a 0.9% NaCl solution at a cell concentration of 10^8^–10^9^ cfu/mL, which was used for pulsed microwave radiation.

### Microwave treatment

The bacterial solution is placed outside of the waveguide in open atmosphere. Microwave power has a 10% error range for every shot-to-shot at higher distance from the waveguide^45^. Therefore, to optimize the electric field outside the waveguide, we used the HFSS simulation model (Fig. 4). From the HFSS plot, it is elucidated that the electric field strength is higher near the virtual cathode and up to 30 cm from this PMR source. By taking 30 cm away (from the waveguide window) as a reference, the receiving antenna was moved closer towards the device with a variation of 1 cm, in order to get a point where the microwave power is the highest. The distance 25 cm away from the window of the waveguide is the point where the microwave power is the highest (~ 33 kW) in this experiment. In addition, this peak power of ~ 33 kW is found to be almost constant along the axial distance from the exit window to the position of the sample. The sample was positioned 25 cm away from the window, where it was exposed to different numbers of PMR discharges (20D, 40D, 60D, and 80D) with interval of 1 min between each shot. The electromagnetic energy “$\mathrm{E}$” for these shots is calculated by:

$$ {\text{E}} = {\text{P}} \times {\text{t}}/2 \times {\text{number}}\;{\text{of}}\;{\text{PMR}}\;{\text{shots}} = (20\;{\text{mJ}},\;40\;{\text{mJ}},\;60\;{\text{mJ}},\;{\text{and}}\;{\text{8}}0\;{\text{mJ}}),\;{\text{respectively}}, $$

where $\mathrm{P}$ ~ 33 kW is the microwave power that reaches the sample and t ~ 60 ns is the pulse duration. Therefore, for the bacterial experiments, 20, 40, 60, and 80 shots are represented in terms of discharges; i.e., 20D, 40D, 60D, and 80D. Further, to better absorb energy, we mounted the bacterial cell solution on an aluminum plate, which was held by a plastic stand, as shown in Fig. 1.

Additionally, the ideal placement of a fixed sample (25 cm away from the waveguide) for microwave radiation was determined with the HFSS code, by observing the highest microwave intensity, i.e., at the quarter-wavelength (λ/4, where λ is the wavelength of the microwave) of the output microwave. The sample was exposed at the positive antinode of the microwave, which was determined by using the WR-284 antenna as the highest strength of the electric field observed up to 30 cm from the waveguide by the HFSS code (Fig. 4).

### Colony forming unit

The cell suspensions were divided into 50-mL sample containers (plastic beakers), which were placed individually for the exposure (25 cm away from the waveguide mounted on the aluminum plate) to pulsed microwave radiation. Following exposure, serial dilutions of 10^7^, 10^6^, 10^5^, 10^4^, and 10^3^ cfu/mL were prepared. The samples were mixed thoroughly, and 100 μL of each microwave-exposed solution was transferred and spread uniformly on Luria–Bertani agar (*E. coli*) or tryptic soy agar (*S. aureus*) culture medium in a standard Petri dish (90 mm). These samples were then sealed and incubated at 37 °C for about 12 h to count the cfu. We used the same method as in previous reports of our group^50,51^. In this study, a relative reduction compared to the control sample was used to represent the inactivation efficacy, where the control cfu was defined as one (unit) for normalization.

### Morphological analysis of bacterial cells

Morphological analyses were conducted using a scanning electron microscope (SEM) (JSM 7001F, JEOL, Tokyo, Japan) to examine the morphology of *E. coli* and *S. aureus* cells. Briefly, the bacterial samples exposed to 40, 60, and 80 PMR discharges were fixed in 1 mL of Karnovsky’s fixative (2% paraformaldehyde and 2% glutaraldehyde) overnight, as described in previous reports^50^. SEM samples were prepared by dehydration in hexamethyldisilazane (HMDS), followed by mounting and coating on glass with carbon tape and examination via SEM.

### Intracellular reactive oxygen species (ROS) and glutathione (GSH) measurement

The total ROS levels inside all bacterial strains were studied using H2DCFDA. After 20, 40 and 80 discharges of PMR, the samples were transferred to a microcentrifuge tube. All exposed samples were washed with PBS, and 500 mL of 10 mM H2DCFDA were added. After incubation for 1 h at 30 °C, the cells were washed with PBS twice. Subsequently, the cells were recovered with PBS at 30 °C for 30 min and analysed at 495/515 (ex./em.) nm using a microplate reader. The mean fluorescence intensity was determined at the corresponding excitation and emission wavelengths.

Intracellular GSH levels were measured using the Glutathione Fluorometric Assay Kit from BioVision according to the manufacturer’s protocol. After 20, 40 and 80 discharges of PMR, the samples were collected and centrifuged at 14,000 rpm for 2 min at 4 °C in cell lysis buffer. The supernatant was used to analyze the GSH level by following the kit protocol. The fluorescent reading was performed at 380/461 (ex/em) nm. GSH concentrations in the samples were calculated by using the standard curve.

### RNA extraction for quantitative real-time PCR

To quantitatively evaluate oxidation-related gene expression, after exposure to different doses of microwave radiation, the total RNA was extracted from treated and untreated *E. coli* samples using an RNeasy Mini Kit and converted to cDNA using reverse transcriptase and random primers (GoScript Reverse Transcription System, Promega, Madison, WI, USA). The same amount of total RNA was used for cDNA synthesis (Take3, Biotek, Winooski, VT, USA) as described in a previous report^52^. The resulting cDNA was used for qPCR analysis (CFX96, Bio-Rad, Hercules, CA, USA) with primers (Macrogen, Seoul, South Korea) of 16 s rRNA (the RNA component of the small subunit, used as the house-keeping gene), SoxS, OxyR, KatG, RpoE, GroES, and DnaK.

The primer sequences used for the oxidative-related mRNA expression in *E. coli* were:

**Table:** 

Genes	Forward primers [5′–3′]	Reverse primers [5′–3′]
SoxS	ATCAGACGCTTGGCGATTAC	ACATAACCCAGGTCCATTGC
OxyR	GGGAAAACTGCTGATGCTG	CGCGGAAGTGTGTATCTTCA
KatG	CGGATCTGGTGTTTGGTTCT	ACAAACTTCTCGTGGGCATC
RpoE	AGTCCCTCCCGGAAGATTTA	ACCTACCGGACAATCCATCCATGA
GroES	TGGCCGTATCCTTGAAAATG	CCGTAGCCATCGTTGAAAAT
DnaK	GAAGAAGCAGGCGACAAACT	TAGCGGCCTTTGTCTTCACCT
16S rRNA	AGAGCAAGCGGACCTCATAA	TTCATGGAGTCGAGTTGCAG

### Detection of DNA damage in *E. coli* cells

The oxidative DNA damage ELISA kit (Cell Biolabs) is a competitive enzyme immunoassay available for rapid detection and quantification of 8-hydroxydeoxyguanosine (8-OHdG), a ubiquitous marker of oxidative stress and a by-product of oxidative DNA damage from cellular DNA samples^53^. The quantity of 8-OHdG in an unknown sample is determined by comparing its absorption with that of a known standard curve. DNA was isolated from the treated and untreated samples using a bacterial DNA isolation kit (Promega) and equal amounts of DNA samples were analyzed for the detection of the 8‐OHdG level using the oxidative DNA damage ELISA kit.

### Statistical analysis

All values are represented as the mean ± SD of the indicated number of replicates. Statistical analysis of the data was performed using Student’s t-test to establish the significance between the data points, and differences were considered significant at *P < 0.05, **P < 0.01, and **P < 0.001. Prism (Graphpad Software Inc., San Diego, CA, USA) and Excel Software (Microsoft Inc., Redmond, WA, USA) were used to compare the groups.

## Results and discussion

### Physical characteristics of PMR

Microwave interactions with biological entities are influenced by multiple factors, such as microwave power and frequency, far-field versus near-field location, exposure duration, polarization, as well as continuous vs pulsed radiation. Pulsed microwave exposure has been shown to have a stronger effect on changes in cell structure and to further enhance cell transformation compared to continuous wave exposure^54,55^. Despite these studies, the effects of PMR on biological systems and the mechanism responsible for the observed biological effects remain uncertain. Thus, in this work, a PMR device based on a pulsed microwave generator was used to investigate its effects on bacterial cells. We first characterized the physical properties, such as diode voltage, diode current, microwave envelope signal, microwave discharge signal, and dominant frequency of the device, using a specially designed microwave receiving antenna, as shown in Fig. 1. The diode voltage and diode current had peak values of 270 kV and 10 kA, as measured by C-dot and B-dot probes, respectively, which resided in the drift tube (Fig. 2a). At each trigger shot, the microwave generated from the virtual cathode propagated toward the antenna. The horn antenna measured the microwave signal, which was positioned 25 cm away from the acryl window of the waveguide (Fig. 2b). The amplitude of different oscillating frequencies from the virtual cathode was measured using the fast Fourier transform method shown in Fig. 2c. The virtual cathode oscillation and the electron reflection between the real and virtual cathodes have different frequencies, but the major oscillating frequency was determined to be 3.5 GHz and the total microwave power generated at the virtual cathode position was approximately 674 MW. Due to the power loss, the microwave power approaching the horn antenna position was ~ 33 kW, in which biological samples were placed, as shown in Fig. 2a. Here, the electric field strength, E_max_, could be estimated to be 12 kV/cm from Poynting power density S, represented by S = E_max_^2^/2*u*_*o*_c, reached at the interaction area A = 2.0 cm^2^ at the sample in this experiment. Based on this, the time-averaged microwave power, P_avg_, could be expressed by P_avg_ = SA, where P_avg_ = 33 kW, *u*_*o*_ = 4π × 10^–7^ T m/s is the magnetic permeability in vacuum, and c = 3.0 × 10^8^ m/s is the speed of light. The thickness of PBS in the beaker is 10 mm, in which the field intensity is resonated inside the PBS caused by the side walls. As shown in the HFSS results, the electric field intensity has a peak distribution of 8 kV/cm at the mid-position between the beaker side walls. Even though there is an absorption loss in power intensity in the PBS solution, it would be passed about 37% through the walls. However, the reflected PMR would be resonated inside the PBS solution, which results in high electric field intensity of 8 kV/cm at the mid-position of the interior beaker tube region. The power density is then estimated to be S = 17 kW/cm^2^ in this experiment.

Continuous microwave radiation causes an increase in temperature of the system and subsequent effects on the bacterial cells. Therefore, in this study, we used pulsed power microwaves with very short pulse duration (60 ns), with interval of three minutes relaxation time, to avoid as much as possible the thermal effect of the microwaves. The temperature of the sample is measured after different microwave exposure, i.e., after 40, 60, and 80 discharges. The temperature of the saline does not show any significant change after the exposure (Fig. 2d). Similar results were obtained in previous studies^56–58^.

### In silico analysis of the electron beam dynamics for virtual cathode formation

To verify the measured physical characteristics of PMR, we investigated the dynamics of the electron beam and the formation of the virtual cathode. Figure 3a shows the diode design of the axial vircator used for the computer simulations. We applied the three-dimensional particle-in-cell (3D PIC) MAGIC code, which simulates as closely as possible the actual operation of the drift tube^45,46^. In the simulation settings, the cathode with diameter of 10 cm was placed at z = 210 mm, and the anode at z = 220 mm, and thus the anode–cathode gap in the device was 10 mm. The real-time incremental behavior of the electron beams, emitted from the cathode in the diode region between the real and virtual cathode, was investigated through numerical simulation. Figure 3b shows the distributions of the electrons in the diode region at 4, 6, and 20 ns after diode breakdown. After 4 ns of diode breakdown, the electron beams began to propagate axially to the drift tube, and most electrons accumulated behind the meshed anode to form the virtual cathode. Some electrons were also observed to be reflected back towards the real cathode due to the virtual cathode formation over time. The electron behavior inside the diode region showed similar characteristics to previous results using MAGIC^45^.

Additionally, to determine the magnitude of the electric field outside the waveguide, i.e., where the samples were kept for microwave exposure, we applied HFSS software^49^. First, an air box with a length of 100 cm was designed, starting from the virtual cathode with diameter of 10 cm. The magnitude of the electric field (Fig. 4a) is found to be as high as ~ 70 kV/cm inside the waveguide and downstream at a distance of ~ 5 cm from the acrylic window, and starts to decrease as it propagates farther downstream from the acrylic window. Furthermore, we also investigated the distribution of the electric and magnetic field in transverse magnetic TM_01_ mode (subscript 0 denotes no radial node and 1 represents polar angle dependence of cosθ) with the HFSS code, where the electric vector field diverges radially and the magnetic vector field has been found curled. In addition, the HFSS code shows the electric field distribution in PBS solution filled in falcon tube (Fig. 4b). This results shows the reflection of PMR by falcon tube surface, which eventually leads to a power loss and induced an electric field of 8 kV/cm. Also, the electric field distribution inside the PBS solution has a resonant patterns along the vertical direction of the test tube. Hence, it might be possible that the electric field of 8 kV/cm generated by PMR in PBS solution induced oxidative stress and intracellular ROS in bacteria.

### Inactivation of *E. coli* and *S. aureus* after pulsed microwave irradiation

To determine the desired location of sample fixation, we had to obtain the point where the intensity of the microwaves is the highest from the HFSS code. From the HFSS results (Fig. 4), it is clear that the electric field increases near the PMR source. At a distance up to 25 cm from the virtual cathode source region inside the waveguide, the electric field is simulated to be almost equal to ~ 70 kV/cm, from both the HFSS and MAGIC codes. The measured electric field obtained at the sample position has been found to be 12 kV/cm (Fig. 4a) from its power density ~ 17 kW/cm^2^ in this experiment. In addition, the electric field in the PBS solution at the sample position has been simulated to be 8 kV/cm (Fig. 4b). This electric field would be sufficient to observe the effect of microwaves on bacteria.

**Figure 5:**
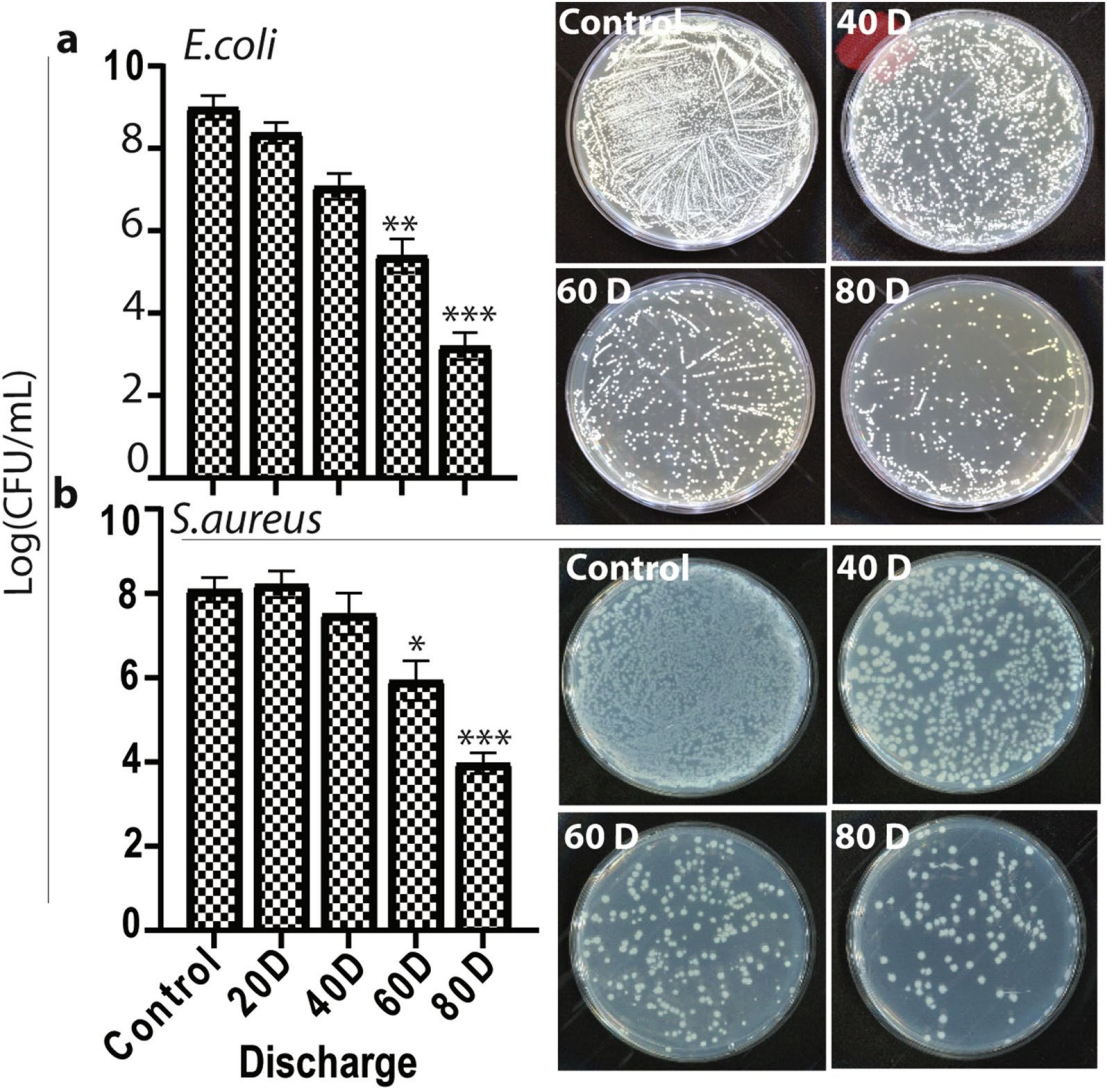
Inactivation of (**a**) *E. coli* and (**b**) *S. aureus* bacterial strains after various doses of microwave radiation. All values are expressed as mean ± SD in triplicate. Student’s t-test was performed for the statistical analysis (*P < 0.05, **P < 0.01, and ***P < 0.001).

To evaluate the influence of microwave radiation on bacterial inactivation, we applied cell suspensions of 10^8^ to 10^9^ colony forming unit/mL (cfu/mL)) of *S. aureus* and *E. coli* bacterial strains (Fig. 5), which were exposed to different numbers of microwave pulses with a power density of ~ 17 kW/cm^2^, i.e., 20, 40, 60, and 80 discharge, represented as 20D, 40D, 60D, and 80D, respectively. The viable counts in both cell suspensions were found to decrease dramatically upon increasing the discharges. 20D did not significantly reduce the viable count compared to the control sample, but at 40D, the viable counts were reduced by about 2-log in *E. coli* and by 1-log in the *S. aureus* cell suspensions. Furthermore, treatment at 60D resulted in an approximate 4-log and 2-log reduction in the viable counts of *E. coli* and *S. aureus*, respectively, compared to the control sample, while 80D resulted in an approximate 6-log reduction in *E. coli* and 4-log reduction in *S. aureus*. Thus, PMR of 40D or more was highly efficient for microbial inactivation. Because the cell wall of gram-positive bacteria is generally much thicker and stronger than that of gram-negative bacteria, *S. aureus* is more resistant to microwave radiation than *E. coli*^59^.

### Effect of microwave radiation on the disruption of the surface structure of bacterial cells

Different cells, organs, and tissues of biological entities have varying dielectric properties; thus, they are affected differently by microwave radiation due to the presence of extra- and intracellular polar molecules, such as lipids, proteins, carbohydrates, DNA, and water. When electromagnetic waves penetrate biological materials, linear momentum (vibrational energy) is generated in the polar molecules, thereby heating up the intra- and extracellular fluids through the transfer of vibrational energy. In this way, radiation energy is converted into thermal energy. However, it remains unclear whether non-thermal effects of microwaves contribute to this. The thermal effects generated by vibrational energy alone cannot explain the manner in which the microwaves affect biological systems. Furthermore, PMR has been postulated to result from a direct stabilizing interaction of the electric field with specific (polar) molecules in cells that are not related to a macroscopic temperature effect^60^. Chen et al. observed the effect of PMR on the killing of microorganisms by altering the cell shape and causing leakage of intracellular proteins or DNA^61^. Nevertheless, it remains unresolved how these morphological changes influence the intracellular cascade during bacterial cell death. Therefore, we examined the surface structure and the intracellular molecules of microwave-irradiated cells.

**Figure 6:**
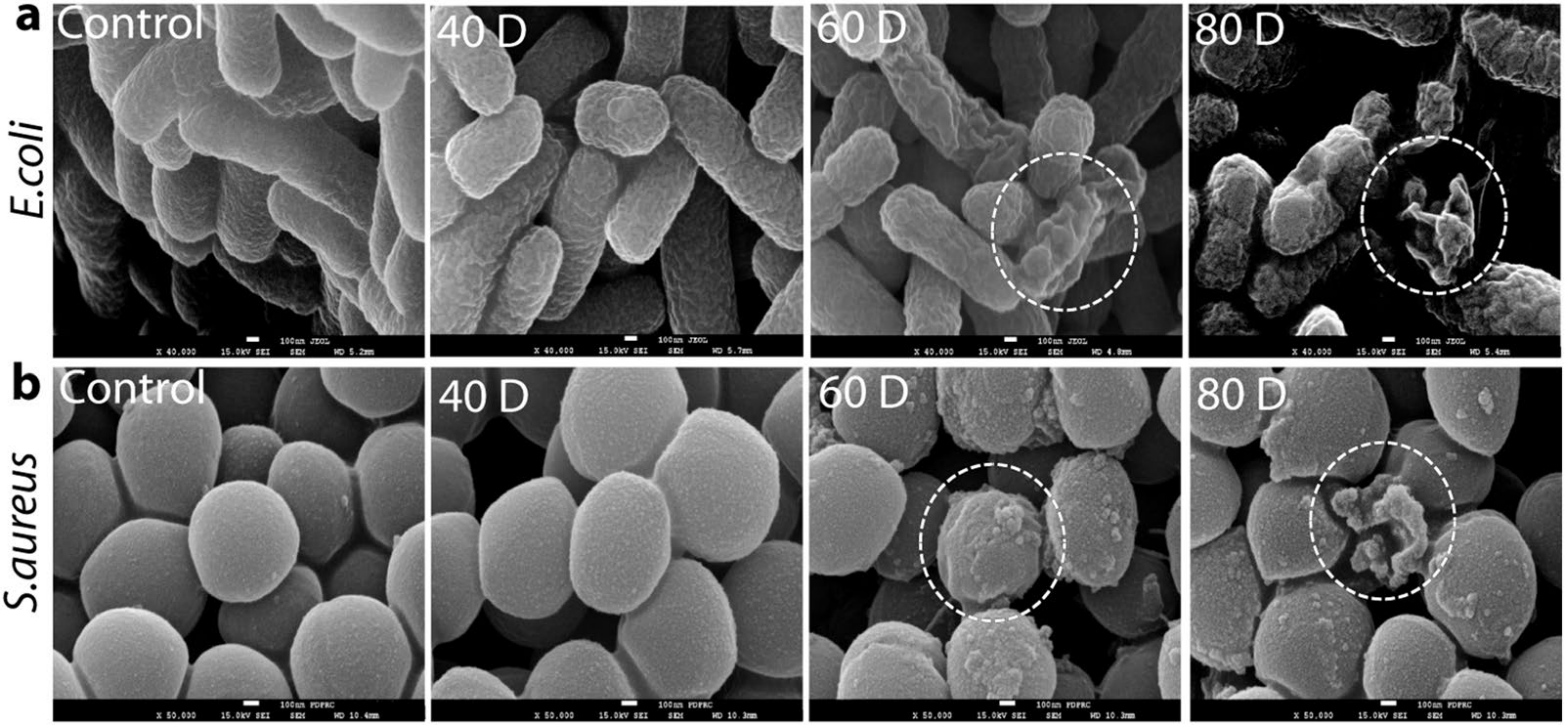
Analysis of cell morphology of (**a**) *E. coli* and (**b**) *S. aureus* bacterial strains after various discharges of microwave radiation.

For this purpose, untreated cells and cells exposed to 40D, 60D, and 80D were examined using a scanning electron microscope (SEM), and the shapes of their surface structures were compared. We found that untreated *E. coli* and *S. aureus* cells had a smooth surface, but when the number of discharges increased from 40D to 80D, most of the microwave-irradiated cells exhibited severe destruction, as shown in Fig. 6a,b. At 80D (for which the exposed energy is 80 mJ), both microwave-irradiated bacterial cell surfaces appear rough and shrunken. However, with fewer discharges at 40D, both bacterial cells exhibited no severe damage to their surface structures. This suggests that the microwave-irradiated cells remain unlysed at lower discharge numbers, up to 40D, but they are inactivated by the radiation. Hence, radiation more than 40 mJ from the PMR would induce different biological effects by differentially partitioning the ions and altering the proteins and lipids in the membrane structure, caused by the strong electric field.

### Assessment of intracellular reactive species (ROS) and glutathione (GSH) levels

**Figure 7:**
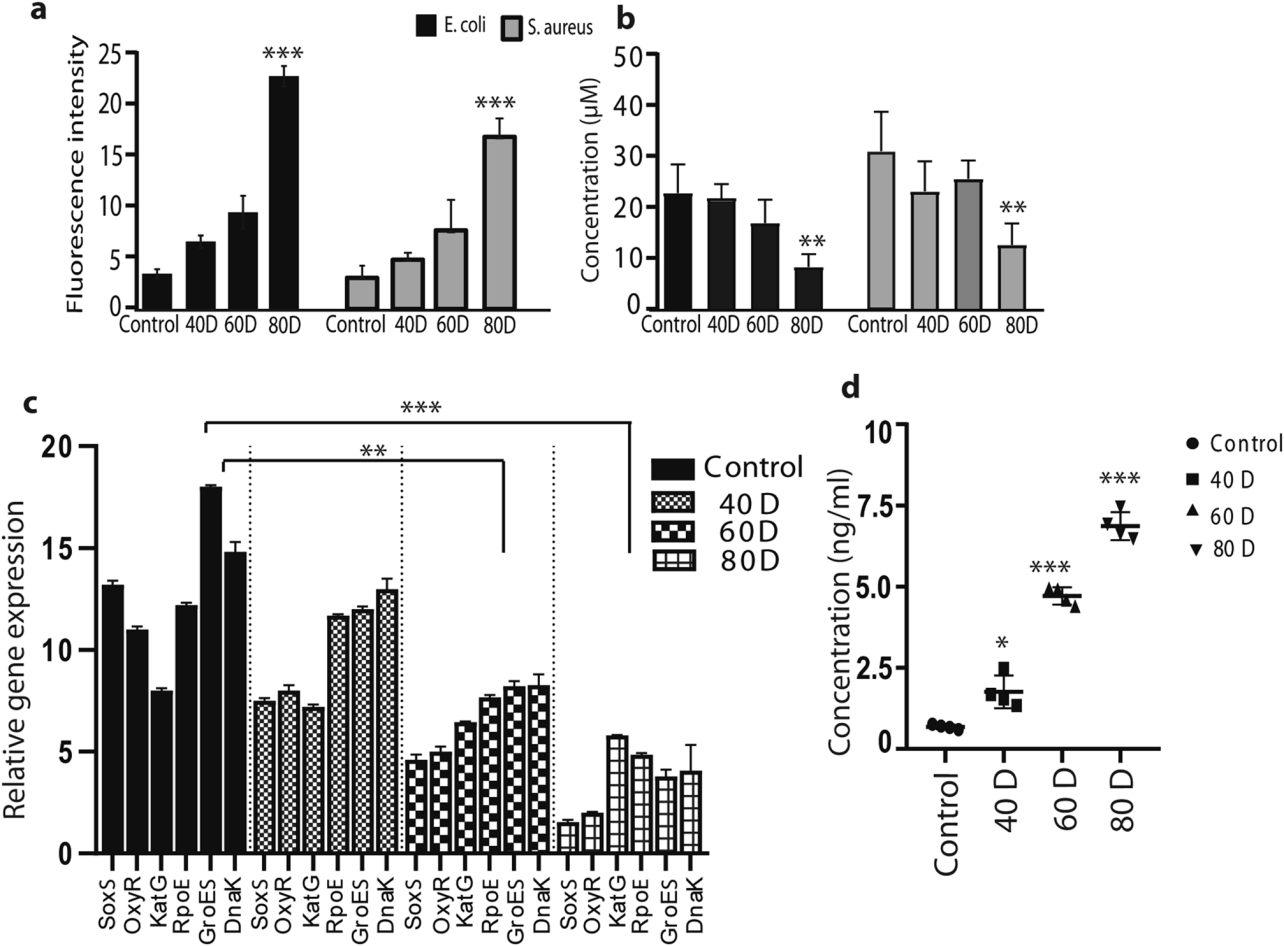
Analysis of (**a**) intracellular RONS levels, (**b**) intracellular glutathione (GSH) level, (**c**) oxidative-related gene expressions, and (**d**) quantification of 8‐hydroxy‐2′‐deoxyguanosine (8‐OHdG), after various discharges of microwave radiation. All values are expressed as mean ± SD in triplicate. Student’s t-test was performed for the statistical analysis (*P < 0.05, **P < 0.01, and ***P < 0.001).

Activation of intracellular ROS plays a very important role in the bactericidal activity, which occurs through a drop in antioxidant machinery, such as a drop in glutathione (GSH) levels and/or by inactivation of glutathione peroxidase 4 (GPX4). Accordingly, we also characterized the potential effect of ROS production upon PMR exposure on the intracellular ROS levels, by measuring changes in the cellular fluorescence intensities of H2DCFDA reagent. This cell-permeable compound dye binds to nuclear DNA and becomes strongly fluorescent upon oxidation. The maximal intensity was significantly increased in both treated bacteria after 80 discharges of microwaves, as compared to the untreated bacterial samples (Fig. 7a). The increasing intracellular ROS levels in response to microwave exposure can further cause a drop in GSH levels, which eventually leads to lethal damage in the bacteria. Interestingly, we also observed a significant decrease in GSH level in both *E. coli* and *S. aureus* after 80 discharges (Fig. 7b). These results suggest that the PMR exposure induces the generation of intracellular ROS and decreases the GSH levels, which contributes to inactivation of both bacteria.

### Analysis of DNA damage and related gene expression in *E. coli*

To date, no available research has demonstrated a solid understanding of how alterations in cell surface cause certain effects in intracellular cell signaling cascades, which lead to cell inactivation. Thus, we tried to elucidate the mechanism of action of PMR on *E. coli* inactivation by analyzing the gene expression. For this purpose, we selected six oxidative stress genes: SoxS (regulation of superoxide response regulon), OxyR (oxygen regulated gene), KatG (catalase), RpoE (DNA-dependent RNA polymerase), GroES (heat-shock gene), and DnaK (chaperone protein DnaK). These proteins perform regulatory functions under stress conditions and help to protect the DNA through antioxidant defense mechanisms^62–65^.

The redox-sensitive transcription factors of the -SoxS, OxyR, KatG, GroES and DnaK genes are related to oxidative stress, while GroES and DnaK are involved in cellular homeostasis^66,67^. Failure of the antioxidant defense, such as the GSH machinery, under high oxidative stress may lead to inhibition of these these genes, which eventually leads to DNA damage^50^.

Higher microwave discharge numbers (80D) led to greater inhibition of the gene expression in PMR-radiated *E. coli* cells (Fig. 7c) compared with 40D and 60D exposure. Indeed, oxidative stress may pass beyond the tolerated threshold under this high exposure level, resulting in the collapse of the antioxidant defense machinery. To understand the extent of DNA damage upon collapse of this machinery, we determined the effects of microwave radiation on genomic DNA damage in *E. coli* through an enzyme-linked immunosorbent assay (ELISA) technique, which was utilized to demonstrate oxidative DNA damage markers (Fig. 7d). We again found that higher discharge numbers, such as 60D and 80D, amplify the level of 8-OHdG, which is the most representative biomarker of oxidative damage to DNA^68,69^, as shown in Fig. 7d.

In summary, this study elucidates the interaction of a broad range of PMR on two different bacterial strains, *E. coli* and *S. aureus*, which cause various community- and hospital-acquired infections. We believe that bacterial inactivation is caused by the chemical breakdown of biological molecules due to the ionization energy^70–72^. Our results show that a certain dose of PMR with electric filed strength 8 kV/cm in this experiment induces significant *E. coli* and *S. aureus* cell mortality and causes changes in cell morphology in both bacteria, cultured in PBS solution. After exposure to microwave radiation, we observed that microwaves created oxidative stress-mediated DNA damage, which might be responsible for bacterial inactivation. Additionally, we measured the transcriptional levels of SoxS, OxyR, KatG, RpoE, GroES, and DnaK in microwave-irradiated *E. coli*. When *E. coli* was exposed to PMR at different discharge numbers, the transcriptional gene expression levels of SoxS, OxyR, KatG, RpoE, GroES, and DnaK were reduced. Higher numbers of discharges, such as 60D and 80D, showed inhibitory effects on gene expression, compared with lower doses, such as 40D. This study clearly shows that a higher discharge number results in greater stress on cells, leading to bacterial inactivation through inhibition of the antioxidant machinery, which eventually damages the membrane protein repair chaperone as well as the DNA repair cascade^73,74^. Furthermore, we analyzed the level of 8-OHdG by the ELISA technique after microwave irradiation. Higher numbers of PMR discharges led to more DNA damage. Overall, this study reveals the action of PMR electric field 8 kV/cm on bacterial systems. These findings may aid in implementing PMR in industrial applications.

## Conclusion

In this study, we experimentally evaluated the output characteristics of a non-thermal pulsed microwave radiation (PMR) device and verified the experimental results in silico. In addition, we applied microwave radiation to two different bacterial strains, the gram-negative *Escherichia coli* and gram-positive *Staphylococcus aureus,* using a PMR device (600 kV, 88 kA, 60 ns) and we examined its mode of action. The electric field of 8 kV/cm generated by PMR in PBS with power density of 17 kV/cm^2^ at the sample position directly interacts with specific (polar) molecules on the bacterial cell surface, causing changes in cell morphology, which further leads to failure of the intracellular oxidative defense machinery and DNA damage-mediated inactivation of bacterial cells. Overall, this study elucidates the mechanisms of PMR-specific effects on bacterial cells and their biomolecules, which can help establish safety standards for PMR exposure without thermal effects on different organisms.

## References

[1] Plazas-Tuttle J, Das D, Sabaraya IV, Saleh NB (2018). Harnessing the power of microwaves for inactivating *Pseudomonas aeruginosa* with nanohybrids. Environ. Sci. Nano.

[2] Wang C, Hu X, Zhang Z (2019). Airborne disinfection using microwave-based technology: Energy efficient and distinct inactivation mechanism compared with waterborne disinfection. J. Aerosol Sci..

[3] Letellier M, Budzinski H (1999). Microwave assisted extraction of organic compounds. Analusis.

[4] Welt B, Tong C, Rossen J, Lund D (1994). Effect of microwave radiation on inactivation of *Clostridium sporogenes* (PA 3679) spores. Appl. Environ. Microbiol..

[5] Rougier C, Prorot A, Chazal P, Leveque P, Leprat P (2014). Thermal and nonthermal effects of discontinuous microwave exposure (2.45 GHz) on the cell membrane of *Escherichia coli*. Appl. Environ. Microbiol..

[6] Jacob J, Chia L, Boey F (1995). Thermal and non-thermal interaction of microwave radiation with materials. J. Mater. Sci..

[7] Kozempel MF, Annous BA, Cook RD, Scullen OJ, Whiting RC (1998). Inactivation of microorganisms with microwaves at reduced temperatures. J. Food Prot..

[8] Shin JK, Pyun YR (1997). Inactivation of *Lactobacillus plantarum* by pulsed-microwave irradiation. J. Food Sci..

[9] Woo I-S, Rhee I-K, Park H-D (2000). Differential damage in bacterial cells by microwave radiation on the basis of cell wall structure. Appl. Environ. Microbiol..

[10] Myint P (2018). Application of gamma irradiation knowledge in tissue sterilisation: Inactivation of malaria parasite. Cell Tissue Bank..

[11] Harrell CR, Djonov V, Fellabaum C, Volarevic V (2018). Risks of using sterilization by gamma radiation: The other side of the coin. Int. J. Med. Sci..

[12] Misra N, Tiwari B, Raghavarao K, Cullen P (2011). Nonthermal plasma inactivation of food-borne pathogens. Food Eng. Rev..

[13] Weisbrod U, Bücker H, Horneck G, Kraft G (1992). Heavy-ion effects on bacteria spores: The impact parameter dependence of the inactivation. Radiat. Res..

[14] Lacombe A (2015). Atmospheric cold plasma inactivation of aerobic microorganisms on blueberries and effects on quality attributes. Food Microbiol..

[15] Sarangapani C, O'Toole G, Cullen PJ, Bourke P (2017). Atmospheric cold plasma dissipation efficiency of agrochemicals on blueberries. Innov. Food Sci. Emerg. Technol..

[16] Baltschukat K, Horneck G (1991). Responses to accelerated heavy ions of spores of *Bacillus subtilis* of different repair capacity. Radiat. Environ. Biophys..

[17] Decareau RV, Kenyon EM (1970). Microwave energy in food processing applications. Crit. Rev. Food Sci. Nutr..

[18] Beszédes S, László Z, Horváth ZH, Szabó G, Hodúr C (2011). Comparison of the effects of microwave irradiation with different intensities on the biodegradability of sludge from the dairy-and meat-industry. Biores. Technol..

[19] Özbek B, Dadali G (2007). Thin-layer drying characteristics and modelling of mint leaves undergoing microwave treatment. J. Food Eng..

[20] Kothari V, Patadia M, Trivedi N (2011). Microwave sterilized media supports better microbial growth than autoclaved media. Res. Biotechnol..

[21] Kakita Y (1995). Inactivation of *Lactobacillus bacteriophage* PL-1 by microwave irradiation. Microbiol. Immunol..

[22] Gorny RL (2007). Viability of fungal and actinomycetal spores after microwave radiation of building materials. Ann. Agric. Environ. Med..

[23] Fung DY, Cunningham F (1980). Effect of microwaves on microorganisms in foods. J. Food Prot..

[24] Vela G, Wu J (1979). Mechanism of lethal action of 2450-MHz radiation on microorganisms. Appl. Environ. Microbiol..

[25] Tanner J, Romero-Sierra C, Davie S (1967). Non-thermal effects of microwave radiation on birds. Nature.

[26] Gubler H, Hiller M (1984). The use of microwave FMCW radar in snow and avalanche research. Cold Reg. Sci. Technol..

[27] Osepchuk JM (1984). A history of microwave heating applications. IEEE Trans. Microw. Theory Tech..

[28] Balmori A (2009). Electromagnetic pollution from phone masts. Effects on wildlife. Pathophysiology.

[29] Tabuse K (1998). Basic knowledge of a microwave tissue coagulator and its clinical applications. J. Hepatobiliary Pancreat. Surg..

[30] Sterzer F (2002). Microwave medical devices. IEEE Microw. Mag..

[31] Grenier K (2013). Recent advances in microwave-based dielectric spectroscopy at the cellular level for cancer investigations. IEEE Trans. Microw. Theory Tech..

[32] Chandra R, Zhou H, Balasingham I, Narayanan RM (2015). On the opportunities and challenges in microwave medical sensing and imaging. IEEE Trans. Biomed. Eng..

[33] Andryieuski A, Kuznetsova SM, Zhukovsky SV, Kivshar YS, Lavrinenko AV (2015). Water: Promising opportunities for tunable all-dielectric electromagnetic metamaterials. Sci. Rep..

[34] Rahman M, Lahri R, Ahsan S, Thanou M, Kosmas P (2020). Assessing changes in dielectric properties due to nanomaterials using a two-port microwave system. Sensors.

[35] Friedman J, Kraus S, Hauptman Y, Schiff Y, Seger R (2007). Mechanism of short-term ERK activation by electromagnetic fields at mobile phone frequencies. Biochem. J.ournal.

[36] Burlaka A (2013). Overproduction of free radical species in embryonal cells exposed to low intensity radiofrequency radiation. Exp. Oncol..

[37] Lu YS, Huang BT, Huang YX (2012). Reactive oxygen species formation and apoptosis in human peripheral blood mononuclear cell induced by 900 MHz mobile phone radiation. Oxid. Med. Cell Longev..

[38] Zmyślony M, Politanski P, Rajkowska E, Szymczak W, Jajte J (2004). Acute exposure to 930 MHz CW electromagnetic radiation in vitro affects reactive oxygen species level in rat lymphocytes treated by iron ions. Bioelectromagnetics.

[39] Poulletier de Gannes F (2011). Effect of exposure to the edge signal on oxidative stress in brain cell models. Radiat. Res..

[40] Gläser K (2016). Effect of radiofrequency radiation on human hematopoietic stem cells. Radiat. Res..

[41] Yakymenko I (2016). Oxidative mechanisms of biological activity of low-intensity radiofrequency radiation. Electromagn. Biol. Med..

[42] Giuliani L, Soffritti M (2010). Non-thermal Effects and Mechanisms of Interaction Between Electromagnetic Fields and Living Matter.

[43] Granatstein VL, Alexeff I (1987). High-Power Microwave Sources.

[44] Miller R (2012). An Introduction to the Physics of Intense Charged Particle Beams.

[45] Mumtaz S (2019). Enhancement in the power of microwaves by the interference with a cone-shaped reflector in an axial vircator. Results Phys..

[46] Jeon W (2006). Output characteristics of the high-power microwave generated from a coaxial vircator with a bar reflector in a drift region. IEEE Trans. Plasma Sci..

[47] Mumtaz S (2018). Enhancing the power of high power microwaves by using zone plate and investigations for the position of virtual cathode inside the drift tube. Phys. Plasmas.

[48] Davis H, Bartsch R, Thode L, Sherwood E, Stringfield R (1985). High-power microwave generation from a virtual cathode device. Phys. Rev. Lett..

[49] ANSYS HFSSTM, see www.ansys.com/hfss, accessed 23rd September 2013.

[50] Shaw P (2018). Bacterial inactivation by plasma treated water enhanced by reactive nitrogen species. Sci. Rep..

[51] Park JH (2015). A comparative study for the inactivation of multidrug resistance bacteria using dielectric barrier discharge and nano-second pulsed plasma. Sci. Rep..

[52] Na YH (2015). Production of nitric oxide using a microwave plasma torch and its application to fungal cell differentiation. J. Phys. D Appl. Phys..

[53] Kumar N (2016). The action of microsecond-pulsed plasma-activated media on the inactivation of human lung cancer cells. J. Phys. D Appl. Phys..

[54] Diem E, Schwarz C, Adlkofer F, Jahn O, Rüdiger H (2005). Non-thermal DNA breakage by mobile-phone radiation (1800 MHz) in human fibroblasts and in transformed GFSH-R17 rat granulosa cells in vitro. Mutat. Res. Genet. Toxicol. Environ. Mutagen..

[55] Czerska EM, Elson EC, Davis CC, Swicord ML, Czerski P (1992). Effects of continuous and pulsed 2450-MHz radiation on spontaneous lymphoblastoid transformation of human lymphocytes in vitro. Bioelectromagnetics.

[56] Mumtaz S (2020). Pulsed high-power microwaves do not impair the functions of skin normal and cancer cells in vitro: A short-term biological evaluation. J. Adv. Res..

[57] Guo C, Wang Y, Luan D (2020). Non-thermal effects of microwave processing on inactivation of *Clostridium sporogenes* inoculated in salmon fillets. LWT.

[58] Chen Z (2017). Evaluation of the possible non-thermal effect of microwave radiation on the inactivation of wheat germ lipase. J. Food Process Eng..

[59] Wang L, Hu C, Shao L (2017). The antimicrobial activity of nanoparticles: present situation and prospects for the future. Int. J. Nanomed..

[60] Herrero MA, Kremsner JM, Kappe CO (2008). Nonthermal microwave effects revisited: on the importance of internal temperature monitoring and agitation in microwave chemistry. J. Org. Chem..

[61] Chen W, Hang F, Zhao J, Tian F, Zhang H (2007). Alterations of membrane permeability in *Escherichia coli* and *Staphylococcus aureus* under microwave. Wei Sheng Wu Xue Bao Acta Microbiol. Sin..

[62] Kilstrup M, Jacobsen S, Hammer K, Vogensen FK (1997). Induction of heat shock proteins DnaK, GroEL, and GroES by salt stress in *Lactococcus lactis*. Appl. Environ. Microbiol..

[63] Wang A, Crowley DE (2005). Global gene expression responses to cadmium toxicity in *Escherichia coli*. J. Bacteriol..

[64] Mongkolsuk S, Helmann JD (2002). Regulation of inducible peroxide stress responses. Mol. Microbiol..

[65] Hiratsu K, Amemura M, Nashimoto H, Shinagawa H, Makino K (1995). The rpoE gene of *Escherichia coli*, which encodes sigma E, is essential for bacterial growth at high temperature. J. Bacteriol..

[66] Lin Z, Rye HS (2006). GroEL-mediated protein folding: Making the impossible, possible. Crit. Rev. Biochem. Mol. Biol..

[67] Vatansever F (2013). Antimicrobial strategies centered around reactive oxygen species–bactericidal antibiotics, photodynamic therapy, and beyond. FEMS Microbiol. Rev..

[68] Kumar N (2014). Induced apoptosis in melanocytes cancer cell and oxidation in biomolecules through deuterium oxide generated from atmospheric pressure non-thermal plasma jet. Sci. Rep..

[69] Shaw P, Kumar N, Privat-Maldonado A, Smits E, Bogaerts A (2021). Cold atmospheric plasma increases temozolomide sensitivity of three-dimensional glioblastoma spheroids via oxidative stress-mediated DNA damage. Cancers.

[70] Reisz JA, Bansal N, Qian J, Zhao W, Furdui CM (2014). Effects of ionizing radiation on biological molecules–mechanisms of damage and emerging methods of detection. Antioxid. Redox Signal.

[71] Corre I, Niaudet C, Paris F (2010). Plasma membrane signaling induced by ionizing radiation. Mutat. Res..

[72] Hu Z-P (2012). Metabolomic response of human skin tissue to low dose ionizing radiation. Mol. BioSyst..

[73] Pomposiello PJ, Demple B (2002). Advances in Microbial Physiology.

[74] Kenley RA, Trevor PL, Lan BY (1981). Preparation and thermal decomposition of pernitric acid (HOONO2) in aqueous media. J. Am. Chem. Soc..

